# Mitochondrial DNA Copy Number Changes in Mood Disorders during the Menopausal Transition

**DOI:** 10.1192/j.eurpsy.2025.1536

**Published:** 2025-08-26

**Authors:** C. Meriç Özgündüz, D. Ceylan

**Affiliations:** 1 Psychiatry, Acibadem University Maslak Hospital; 2 Neuroscience, Koç University Graduate School of Health Sciences; 3 Psychiatry, Koç University Faculty of Medicine, İstanbul, Türkiye

## Abstract

**Introduction:**

A heightened susceptibility to mood disorders among women at specific junctures throughout their reproductive lifetime has been reported. Previous studies have suggested that the probability of developing Major Depressive Disorder (MDD) during the menopausal transition is amplified from 2 to 4 times when contrasted with both premenopausal and postmenopausal periods and also increased mood symptom severity in the menopausal transition in bipolar disorder (BD) has been cited (Chen *et al.* Arch Womens Ment Health 2017; 20 463-8). A considerable body of evidence suggests that mitochondrial dysfunction plays a crucial role in the pathophysiology of various psychiatric disorders, including MDD and BD. Researchers have proposed that the mitochondrial DNA copy number (mtDNAcn) may serve as an essential marker of mitochondrial health, which is the ratio of mitochondrial and nuclear DNA (Malik *et al*. Mitochondrion 2013; 13 481-92).

**Objectives:**

In this review, we aimed to compile and present existing data regarding mtDNAcn alterations in women during the menopausal transition by comparing the mtDNAcn numbers of the women in premenopausal, perimenopausal and postmenopausal periods thereby establishing a foundation for future research on the origins of these conditions.

**Methods:**

A literature search was conducted on the electronic databases PubMed, Cochrane Library, Medline (OVID), Scopus, Web of Science and CINAHL to identify the relevant studies published until 14 October 2024. The search was performed using the following keywords (Menopause OR Premenopause OR Pre-Menopause OR Premenopaus* OR Perimenopause OR Postmenopause OR Post-Menopause OR Postmenopaus*) AND (mitochondrial DNA copy number OR mtDNA copy number) and limited to English language.

**Results:**

According to the search results, 66 publications were reached in total. After detecting and removing duplicate publications, there were 21 publications left. After screening, any of the studies met inclusion criteria for the systematic review, because most of them were conducted in different patient groups and healthy controls in the menopausal transition periods, but the main findings of these studies can be summarized as; mtDNAcn varies between the patients groups and healthy controls in the menopausal transition periods with the conflicting results. Some of the studies found higher mtDNAcn in patient groups such as breast cancer and pelvic organ prolapsus, whereas some of them found lower mtDNAcn numbers in patient groups such as osteopenia and osteoporosis.

**Conclusions:**

Considering above findings, there is mtDNAcn alterations in women during the menopausal transition. These findings may highlight the need for future studies to elucidate the link with mtDNAcn and mood disorders in the menopausal transition period by comparing the women in different menopausal periods.

**Disclosure of Interest:**

None Declared

